# Rapid Induction of Lipid Droplets in *Chlamydomonas reinhardtii* and *Chlorella vulgaris* by Brefeldin A

**DOI:** 10.1371/journal.pone.0081978

**Published:** 2013-12-13

**Authors:** Sangwoo Kim, Hanul Kim, Donghwi Ko, Yasuyo Yamaoka, Masumi Otsuru, Maki Kawai-Yamada, Toshiki Ishikawa, Hee-Mock Oh, Ikuo Nishida, Yonghua Li-Beisson, Youngsook Lee

**Affiliations:** 1 Division of Molecular Life Sciences, POSTECH, Pohang, Korea; 2 POSTECH-UZH Global Research Laboratory, Division of Integrative Biology and Biotechnology, POSTECH, Pohang, Korea; 3 Division of Life Science, Graduate School of Science and Engineering, Saitama University, Saitama, Saitama, Japan; 4 Institute for Environmental Science and Technology, Saitama University, Saitama, Saitama, Japan; 5 Environmental Biotechnology Research Center, Korea Research Institute of Bioscience and Biotechnology (KRIBB), Daejeon, Korea; 6 Department of Plant Biology and Environmental Microbiology, CEA-CNRS-Aix Marseille University, Saint-Paul-Lez-Durance, France; Medical University of South Carolina, United States of America

## Abstract

Algal lipids are the focus of intensive research because they are potential sources of biodiesel. However, most algae produce neutral lipids only under stress conditions. Here, we report that treatment with Brefeldin A (BFA), a chemical inducer of ER stress, rapidly triggers lipid droplet (LD) formation in two different microalgal species, *Chlamydomonas reinhardtii* and *Chlorella vulgaris.* LD staining using Nile red revealed that BFA-treated algal cells exhibited many more fluorescent bodies than control cells. Lipid analyses based on thin layer chromatography and gas chromatography revealed that the additional lipids formed upon BFA treatment were mainly triacylglycerols (TAGs). The increase in TAG accumulation was accompanied by a decrease in the betaine lipid diacylglyceryl *N,N,N*-trimethylhomoserine (DGTS), a major component of the extraplastidic membrane lipids in *Chlamydomonas*, suggesting that at least some of the TAGs were assembled from the degradation products of membrane lipids. Interestingly, BFA induced TAG accumulation in the *Chlamydomonas* cells regardless of the presence or absence of an acetate or nitrogen source in the medium. This effect of BFA in *Chlamydomonas* cells seems to be due to BFA-induced ER stress, as supported by the induction of three homologs of ER stress marker genes by the drug. Together, these results suggest that ER stress rapidly triggers TAG accumulation in two green microalgae, *C. reinhardtii* and *C. vulgaris.* A further investigation of the link between ER stress and TAG synthesis may yield an efficient means of producing biofuel from algae.

## Introduction

Biofuel production from food crops is steadily increasing, resulting in competition for agricultural land between food and fuel production. To retain food sources for an ever-increasing population, it is imperative that new feedstocks for biofuel be identified that can replace fossil fuel [1]. Many microalgae grow rapidly and produce a large amount of biomass, and under certain cultivation conditions, can also store large amounts of oil that can readily be converted into biodiesel [2]. Microalgae have thus been suggested as an alternative source for green renewable energy production [3].

*Chlamydomonas reinhardtii* is a model organism for studying many biological processes, including photosynthesis and flagella function [4]. More recently, it was also used to study microalgal lipid metabolism. This photosynthetic green alga uses sunlight and CO_2_ to produce chemical energy in the form of carbohydrates and lipids, and requires nitrogen to synthesize the proteins necessary for cell growth [4]. Under stress conditions (notably nitrogen depletion), *Chlamydomonas* starts to accumulate significant amounts of neutral lipids (mainly triacylglycerols, TAGs) in distinct cellular organelles called lipid droplets (LDs) [5]–[7]. The fatty acids required for TAG assembly are either synthesized *de novo* or produced from recycled acyl chains from membrane lipids [5]–[7]. Under culture conditions widely used in laboratories, exogenously supplied acetate boosts TAG production *via* the *de novo* pathway. The exact contribution of each pathway to TAG synthesis under specific conditions remains to be elucidated.

Among the many stress conditions, nitrogen starvation is the most effective means of triggering oil accumulation in algae [7]. However, this approach is not ideal because *i)* nitrogen depletion reduces photosynthesis and thus the overall production of biomass and *ii)* at the industrial level, nitrogen removal is energy intensive, time-consuming, and costly [8]. Thus, it would be beneficial to identify methods of inducing oil accumulation in cells that do not involve nitrogen starvation and that can be used to culture many different algae [2].

Brefeldin A (BFA) is a drug well-known for its inhibitory effect on vesicular transport from the endoplasmic reticulum (ER) to the Golgi, which causes retrograde transport of vesicles from the Golgi to the ER [9]. BFA thus disrupts trafficking of newly synthesized and modified proteins and membrane lipids (mainly phospholipids and sterols) to their final destinations, causing unfolded protein accumulation and lipid composition changes [10], [11], all of which are symptoms of ER stress. Notably, the ER stress induced by BFA treatment induces neutral lipid accumulation in *Saccharomyces cerevisiae*, with corresponding increases in the number of cellular LDs [11], [12]. ER stress can be detected in organisms by monitoring the expression levels of ER stress marker genes, including BiP (lumenal binding protein) and SAR1 (secretion associated membrane protein 1).

Here, we report that BFA treatment rapidly induces LD formation in two species of microalgae, *Chlamydomonas reinhardtii* and *Chlorella vulgaris,* and that the lipids induced by BFA treatment are mainly TAGs. Our chemical analysis of lipids suggests that this neutral lipid accumulation is due to perturbation of lipid homeostasis at the ER and other extra-plastidial membranes. In support of this explanation, BFA induces the expression of several ER stress marker gene homologs in *Chlamydomonas reinhardtii*, suggesting that ER stress causes TAG accumulation. Interestingly, the lipid induction by BFA is very fast, and occurs regardless of whether or not nitrogen or acetate is present in the medium, suggesting that the drug induces LD accumulation *via* a pathway independent of that triggered by nutrient starvation.

## Materials and Methods

### Cell Culture

The *Chlamydomonas reinhardtii* strains *CC-503* wild type *mt+* (*cw92*) and *CC-125* wild type *mt+* (*137c*) were obtained from the Chlamydomonas Genetics Center (USA). *Chlamydomonas* strains were cultured in Tris acetate phosphate (TAP) medium [12] at 25°C under continuous light with shaking. *Chlorella vulgaris* cells were obtained from the Biological Resource Center (BRC, Korea) and cultured in BG11 medium [13] under the same conditions as used for *Chlamydomonas*.

### LD Induction by BFA Treatment

Brefeldin A (LC Labs) was dissolved in dimethyl sulfoxide (DMSO) at a concentration of 50 mg mL^−1^ and stored at −20°C until use. An aliquot of this stock solution was added to cell culture media to obtain working concentrations. The same volume of DMSO was added to solvent control samples.

### LD Staining and Observation by Fluorescence Microscopy

LDs in *Chlamydomonas* and *Chlorella* cells were stained with Nile red (Sigma) [14]. The cells were incubated with Nile red at a final concentration of 1 µg mL^−1^ (prepared from a stock solution of 0.1 mg mL^−1^ in acetone) for 30 min in the dark at room temperature (∼25°C) [7]. Stained cells were then observed under fluorescence microscopy (Nikon, Optihot-2, Japan) using optical filters designed for fluorescein isothiocyanate (FITC) and tetramethyl rhodamine iso-thiocyanate (TRITC).

### Quantification of LDs by Fluorescence Spectrophotometry and Flow Cytometry

To further quantify the fluorescence signal, Nile red intensity was measured using a Safire fluorescence spectrophotometer (TECAN, Switzerland) with a 488-nm excitation filter and a 565-nm emission filter. Flow cytometry of *Chlamydomonas* was performed on a FACS Calibur Flow Cytometer (Becton Dickinson). A 10 µL aliquot of 100 µg mL^−1^ Nile red in acetone was added to 5.0×10^6^ cells in 1 mL of TAP medium 30 min before flow cytometry analysis. Twenty thousand cells were analyzed without gating. The red fluorescence of Nile red was plotted on a histogram.

### Measurement of Cell Concentration

Cell growth was monitored by measuring the optical density (OD) at 750 nm using a Safire fluorescence spectrophotometer (TECAN). The absorbance of 750-nm light usually correlates well with the biomass of the culture [15]. Cell number was also counted using a hemocytometer.

### Lipid Extraction and Quantification

Total lipids were analyzed as described in The Plant Organelle Database 2 [16] [Ikuo Nishida, Lipid extraction from Arabidopsis mitochondria, http://podb.nibb.ac.jp/Organellome/bin/findFunctional?status=confirm&method=1&Species=&Keywords=&Category=Biochemical assays]. *CC-503* cells (approximately 5.0×10^7^) were grown in TAP medium containing either BFA or DMSO alone (solvent control) and subjected to lipid extraction. Briefly, cells were harvested by centrifugation at 2,000 *g* for 20 min. The pellets were immersed in 2 mL of boiling isopropanol and heated for 5 min at 80°C to inactivate lipases. After cooling, 2 mL each of methanol and chloroform and 1.24 mL of water were added to the sample and the mixture was vortexed for 5 min. The extract was centrifuged (10 min, 2,000 *g*), the resultant supernatant decanted to a new 10-mL screw-capped glass tube, and the pellet re-extracted with 2 mL of methanol, 1 mL of chloroform, and 0.8 mL of water by vortexing. After centrifugation (10 min, 2,000 *g*), the supernatant was recovered by decantation, combined with the first supernatant, and then washed with 3 mL of 0.9% KCl (w/v) by vigorous shaking. After centrifugation (10 min, 2,000 *g*), the lower layer was recovered and the solvent was evaporated under nitrogen stream. To quantify TAGs, dried lipid residues were dissolved in chloroform and separated on silica gel TLC using a solvent mixture that facilitates the separation of neutral lipids [80/30/1 (v/v/v), hexane/diethylether/acetic acid]. For separation of phospholipids and polar acyl lipids, two-dimensional TLC was performed [first dimension: 160/60/40/20 (v/v/v/v) acetone/toluene/methanol/water; second dimension, 170/25/25/4 (v/v/v/v) chloroform/methanol/acetic acid/water]. Lipid spots were visualized under UV after spraying with 0.01% (w/v) primuline (Sigma) dissolved in acetone: H_2_O (4∶1 v/v). Corresponding lipid bands were recovered from the plate and quantified by gas chromatography (GC) (SHIMADZU GC-2010, HP-INOWAX capillary column, 30 m, 0.25 mm) after being converted to fatty acid methyl esters (FAMEs) *via* an acid-catalyzed transesterification protocol previously described by Li *et al.* 2006.

### RNA Extraction and Quantification

Total RNA was extracted according to the phenol/chloroform method described in the [17], with a few modifications. Total RNA was isolated using RNA extraction buffer (250 mM Tris HCl [pH 9.0], 250 mM NaCl, 50 mM EDTA, 345 mM *p*-aminosalicylic acid, 27 mM triisopropyl naphthalene sulfonic acid, 250 mM ß-mercaptoethanol, 0.024% [v/v] phenol) [18], and cDNA was synthesized by reverse transcription using 4 µg of total RNA. Real-time PCR was performed using primer sets designed to amplify *Chlamydomonas BiP* homologs *CrBiP1* (5′-AGT GAG CCC GTC TTT TAG AAC TT-3′ and 5′-TCT CCT CTG TAC CAC CGT TTT TA-3′), *CrBiP2* (5′- TAT CGC TAG TGC ATT TGT TTG AA-3′ and 5′-GTT GAA GGA AGC AGA ACA AAA GA-3′), or *CrSAR1* (5′-CGA GGA GAT TCA ATT GGG CG-3′ and 5′-CGG TGG GAA TGT CGA TCT TG-3′), according to the information in Phytozome v9.1 at www.phytozome.net. The real-time PCR results were normalized by the level of *RPL17* expression [19].

### Chlorophyll Measurements

Chlorophyll content was measured using the ethanol extraction method [20]. A 1-mL aliquot of culture, at a concentration of 5.0×10^6^ cells mL^−1^, was centrifuged, and the pellet was resuspended and vortexed in 95% ethanol. Cellular debris was removed by centrifugation and chlorophyll *a* and *b* levels in the supernatant were determined by measuring optical absorbance at 648 nm and 664 nm, respectively. Total chlorophyll content was calculated as described previously [20].

## Results

### BFA Treatment Induces LD Formation in the Model Microalga *Chlamydomonas reinhardtii*

In an effort to identify alternative methods of triggering oil accumulation in green microalgae, we evaluated the effects of a well-known vesicle trafficking inhibitor, brefeldin A (BFA), on lipid droplet (LD) formation in *Chlamydomonas reinhardtii*. As an initial test, we incubated a culture of cell wall-less *Chlamydomonas* strain *CC-503* in TAP medium until the mid-log phase (approximately 5.0×10^6^ cells mL^−1^), and then treated the cells with BFA for 4 h at a final concentration of 75 µg mL^−1^. We monitored the occurrence of LDs in BFA-treated cells by staining the samples with a fluorescent indicator dye for lipid bodies, Nile red, and then monitoring them by fluorescence microscopy.

**Figure 1:**
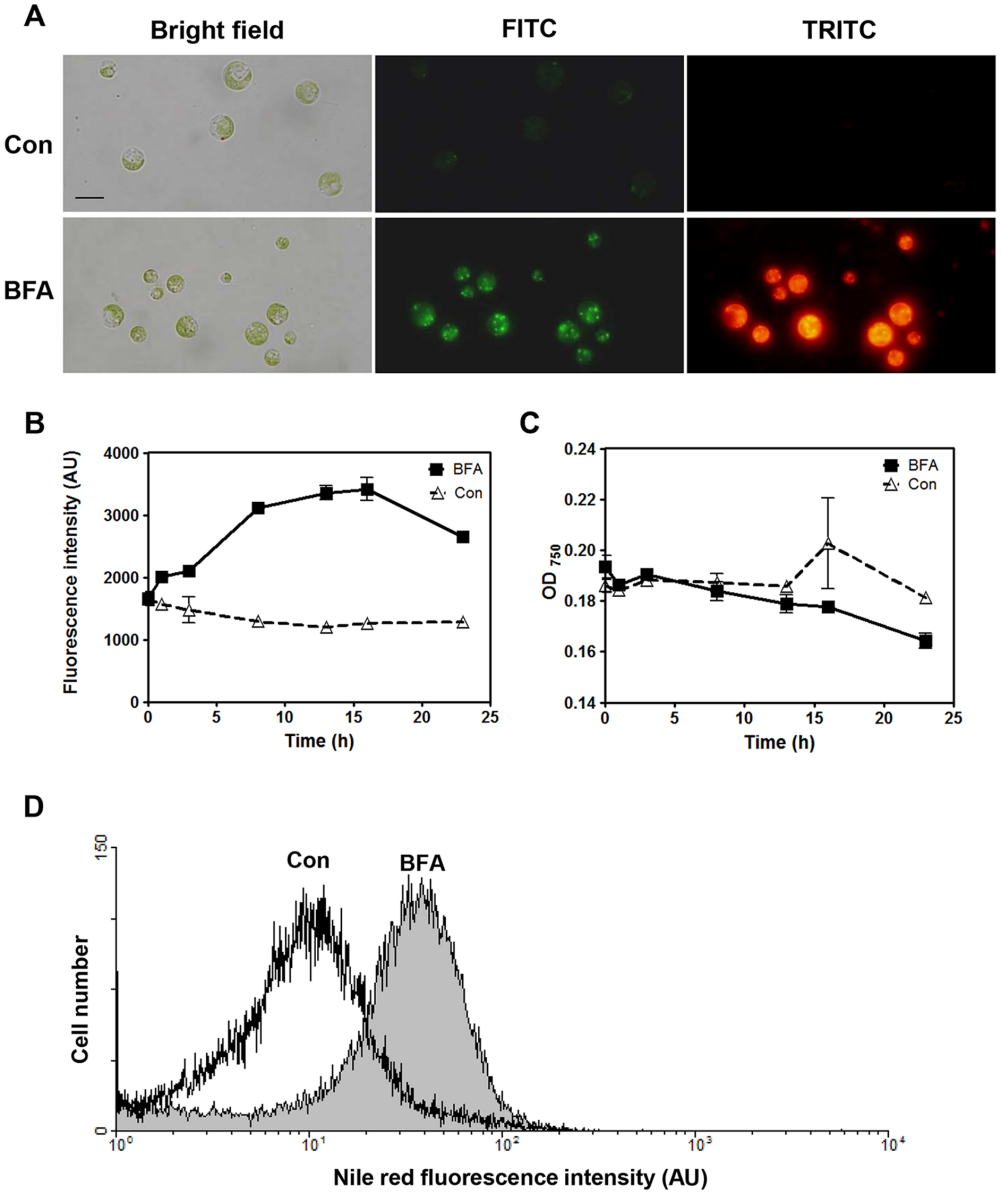
BFA treatment induces lipid droplet (LD) formation in *Chlamydomonas reinhardtii* strain *CC-503*. (A) Images of Nile red-stained LDs in BFA-treated cells. Images were acquired after cells were incubated for 4 h with BFA at a concentration of 75 µg mL^−1^. DMSO was used as a solvent control (Con). Bar = 10 µm. Nile red fluorescence was viewed using either the 465–495 nm excitation/515–555 nm emission channel (FITC, green) or the 540/25 nm excitation/605/55 nm emission channel (TRITC, red). (B) Time-dependent effect of BFA treatment on the fluorescence intensity of LDs stained with Nile red. The fluorescence was quantified using a fluorescence spectrophotometer with a 488-nm excitation filter and a 565-nm emission filter. AU = arbitrary units. (C) Growth of the *Chlamydomonas* culture treated with BFA, measured by reading at OD_750_. (D) Quantification of Nile red fluorescence in individual BFA-treated cells by flow cytometry. We examined 20,000 cells subjected to two different conditions (DMSO solvent control, and BFA at 75 µg mL^−1^). AU = arbitrary units.

Whereas numerous fluorescent LDs were observed in BFA-treated cells, none or only a few were present in control cells (treated only with DMSO, the solvent used for dissolving BFA) (Fig. 1A). LDs were highly fluorescent under either the TRITC or FITC filter set, but they were more clearly distinguishable from the large chloroplast when the FITC filter set was used. Time dependency tests using a TECAN fluorescence spectrophotometer revealed that, in normal TAP medium supplemented with nitrogen (+N) and acetate (+Ac), the addition of BFA rapidly (within 2 h) induced the formation of LDs, as gauged by an increase in Nile red fluorescence (Fig. 1B). The Nile red fluorescence signal plateaued between 8 and 15 h after BFA addition. However, BFA treatment for 8 h did not cause any significant change in total chlorophyll content or chlorophyll *a*/*b* ratios (Fig. S1). Incubation beyond 15 h did not increase the Nile red fluorescence signal, but decreased the reading at OD_750_, a parameter indicative of cell biomass (Fig. 1C), suggesting damage to the cells and a consequent decline in biomass. Thus, for the following experiments, we treated the cells with BFA only for an 8-h period, unless otherwise specified.

To examine the effect of BFA at the cell population level, we analyzed the Nile red fluorescence intensity of 20,000 cells from each group (i.e., treatment and control) using flow cytometry. Many more cells with high fluorescence levels were present in the BFA-treated culture than in the solvent control culture (Fig. 1D), indicating that BFA treatment induced LD accumulation in many cells of the *Chlamydomonas reinhardtii* culture.

We also tested the effect of BFA treatment on LD formation using another strain of *Chlamydomonas reinhardtii, CC-125,* which has a cell wall. To allow BFA to penetrate the cell wall efficiently, the samples were sonicated immediately before BFA was added. LD formation was again tracked by staining with Nile red. As shown in figure S2, BFA induced LD formation, and the extent of LD induction correlated well with the length of time of sonication, suggesting that the more BFA that enters the cells, the more LDs the cells accumulate.

### Dose-dependent and Growth Phase-dependent Effects of BFA Treatment

**Figure 2:**
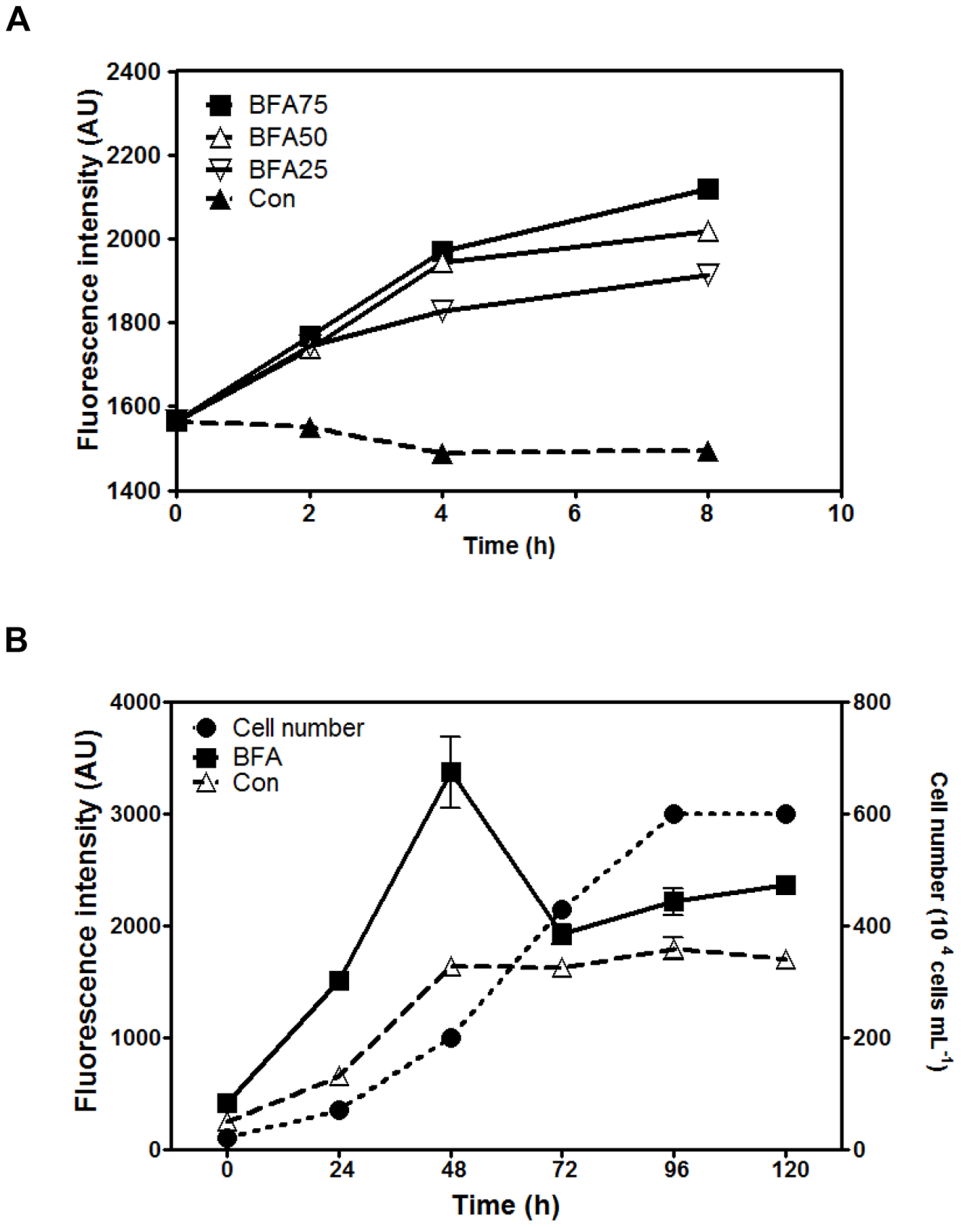
Dose-dependence and growth phase-dependence of the effect of BFA treatment on LD formation in *Chlamydomonas reinhardtii CC-503*. (A) Fluorescence intensity of LDs stained with Nile red in cells treated with different BFA concentrations (25, 50, and 75 µg mL^−1^) for the indicated durations. (B) Nile red fluorescence intensity of LDs in cells treated at different stages of cell culture with either 75 µg mL^−1^ BFA (closed squares) or DMSO (solvent control, Con; open triangles). Cells were grown in normal TAP medium, and at 24-h intervals, the number of cells in culture (closed circles) was analyzed using a hemocytometer, and the 8-h treatment with BFA was started. AU = arbitrary units.

To decipher the mechanisms underlying BFA action, *Chlamydomonas* cells were treated with different concentrations of BFA, ranging from 0 to 75 µg mL^−1^. As shown in figure 2A, levels of Nile red fluorescence were similar at 2 h, but after 8 h, higher levels of Nile red fluorescence were detected in cells treated with higher concentrations of BFA (Fig. 2A). We then tested the effect of BFA treatment on the induction of lipid bodies at different growth phases of the *Chlamydomonas* cell culture. BFA treatment increased Nile red fluorescence at all growth phases, but the drug was most effective when applied at the early log phase (48 h) (Fig. 2B).

### BFA Treatment Induces TAG Accumulation at the Expense of DGTS

**Figure 3:**
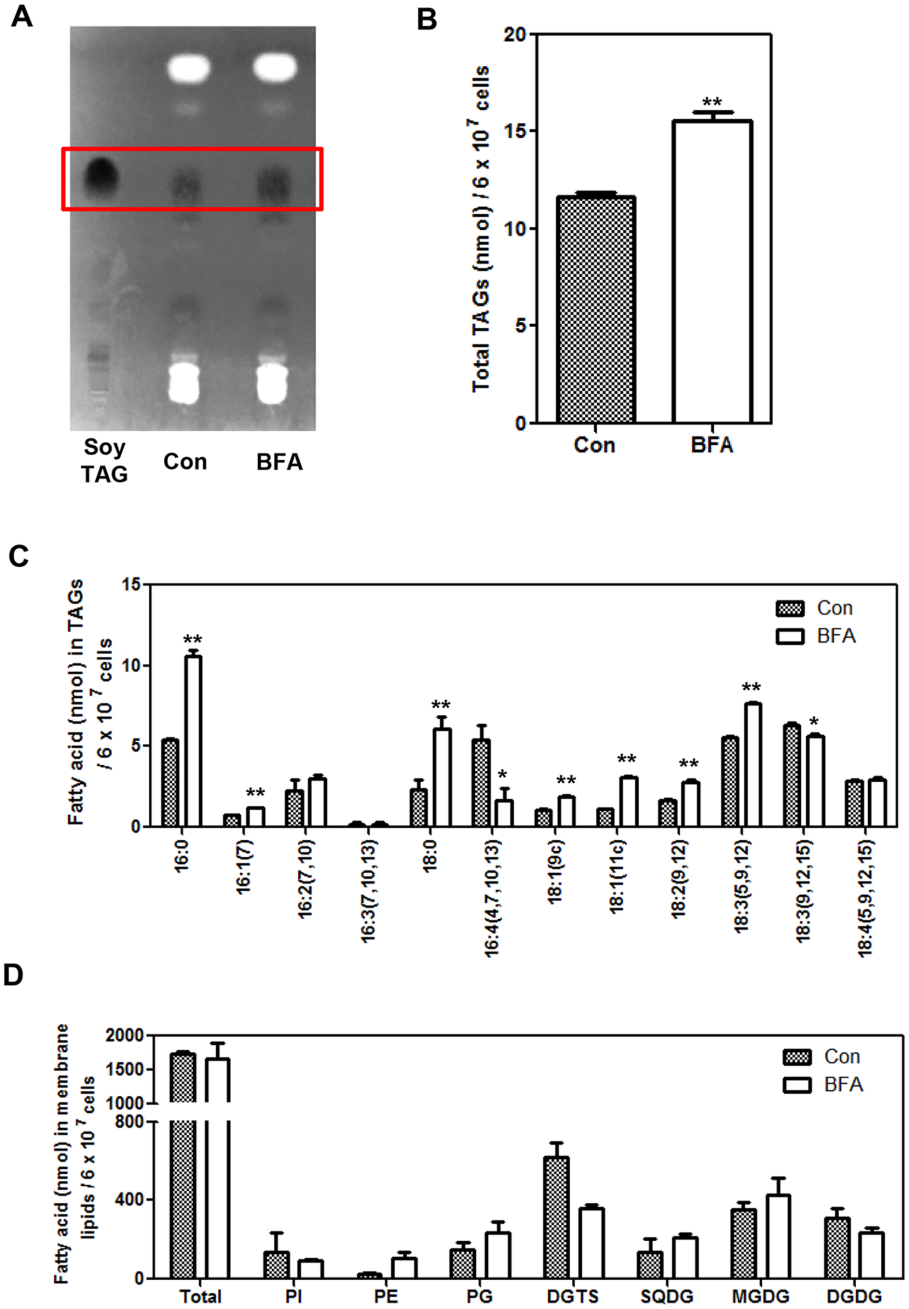
Changes in lipid composition of BFA-treated *Chlamydomonas reinhardtii CC-503*. Cells were grown to the mid-log phase, treated with BFA (75 µg mL^−1^) or DMSO (solvent control, Con) for 8 h, and then lipids were analyzed. (A) Accumulation of TAGs on a TLC plate as revealed by staining with primuline. The bands in the box are TAGs with the same Rf value as soybean TAGs. (B) BFA-treated cells accumulate higher amounts of TAGs than control cells. (C) Comparison of fatty acid compositions of TAGs isolated from BFA-treated and control (Con) cells. In (B) and (C), three replicates were averaged, and the SEs are shown. Significant differences, as determined by Student’s *t*-test, are indicated by asterisks (*P<0.05, **P<0.01). (D) Comparison of the major lipid classes between BFA-treated and control (Con) cells. Averages from two replicate experiments and their standard deviations are shown. PI, phosphatidylinositol; PE, phosphatidylethanolamine; PG, phosphoglyceride; DGTS, diacylglyceroltrimethylhomoserine; SQDG, sulfoquinovosyl-diacylglycerol; MGDG, monogalactosyldiacylglycerol; and DGDG, digalactosyldiacylglycerol.

In *Chlamydomonas reinhardtii*, LDs formed upon nitrogen starvation contain mainly triacylglycerols (TAGs) [21]. To examine if the LDs induced by BFA treatment also contain mainly TAGs, total lipids were extracted from *CC-503* cells after an 8-h treatment with BFA. Analysis of a TLC plate stained with primuline and viewed under UV illumination revealed that more TAGs accumulated in BFA-treated cells than in control cells (Fig. 3A). Recovery of the bands corresponding to the TAGs and quantification by GC confirmed that the concentration of TAGs was 34% greater in the treated cells compared to the control (Fig. 3B).

BFA treatment significantly increased the levels of 16∶0, 16∶1(7), 18∶0, 18∶1(9), 18∶1(11), 18∶2(9,12), and 18∶3(5,9,12) and significantly decreased the levels of 16∶4(4,7,10,13) and 18∶3(9,12,15) in TAGs (Fig. 3C). Especially noteworthy were the increase in 18∶3(5,9,12), the major fatty acid of diacylglycerol-*N,N,N*-trimethylhomoserine (DGTS), a non-plastidial lipid [22]–[25], and the decrease in 16∶4(4,7,10,13) and 18∶3(9,12,15), the two major components of plastidial lipids [25]. These fatty acid profiles indicated that the fatty acid substrates used for BFA-induced TAG assembly were derived mainly from non-plastidial membrane lipids.

### Changes in Lipid Composition Induced by BFA Treatment

To further identify which membrane lipids were converted into TAGs in BFA-treated cells, polar lipid compositions were examined as described in Materials and methods. Total lipids were extracted from cells and major polar lipids were separated by two-dimensional TLC and quantified by GC after derivatization into fatty acid methyl esters.

The amount of total lipids did not differ between BFA-treated and control cells (Fig. 3D), despite a small increase in TAG accumulation in BFA-treated cells (Fig. 3B). TAG content was much lower than that of total membrane lipids (compare the y-axes of Figs. 3C and 3D). DGTS was reduced by over 30% upon BFA treatment, whereas a moderate increase in phosphatidylethanolamine (PE) and a moderate decrease in digalactosyldiacylglycerol (DGDG) were observed (Fig. 3D). The content of other lipids changed much less. The reduction in DGTS by BFA further supports the hypothesis that a portion of recycled acyl chains from the non-plastidial membrane lipids was used for TAG synthesis in BFA-treated cells.

### BFA can Induce TAG Synthesis via Pathways that are Different from Acetate Boosting or Nitrogen Starvation

**Figure 4:**
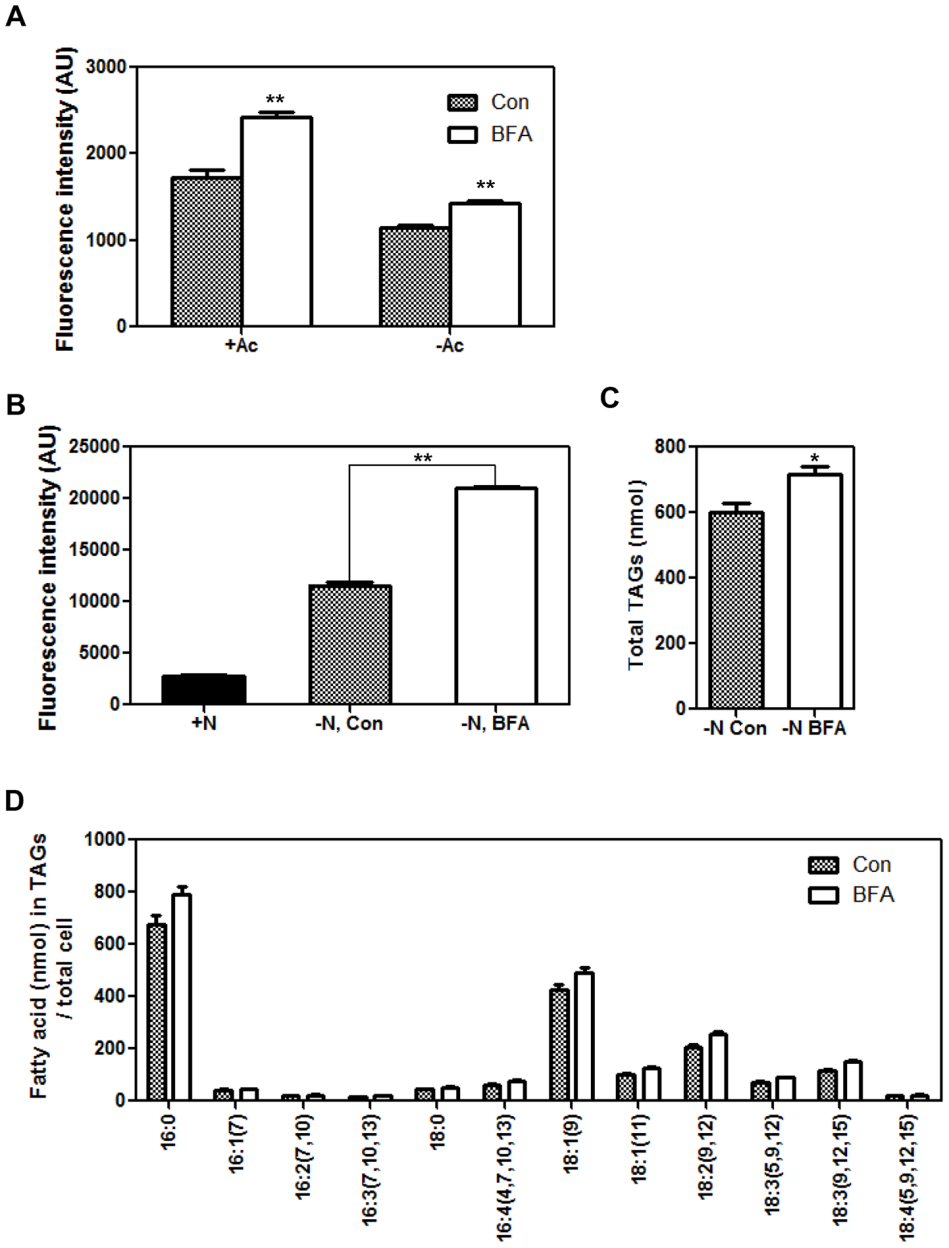
BFA-induced LD formation is independent of the presence of an acetate or nitrogen source in the medium. (A) Nile red fluorescence from *CC-503* cells grown in normal TAP (+N, +Ac) medium, transferred to medium with or without acetate, and incubated in continuous light. After an 8-h incubation, Nile red fluorescence was measured for control (Con: DMSO solvent control) and BFA-treated cells using a fluorescence spectrophotometer equipped with a 488-nm excitation filter and a 565-nm emission filter. AU = arbitrary units. (B) Nile red fluorescence of *CC-503* cells grown in nitrogen-replete or nitrogen-deficient medium for 1 day, and then treated with BFA for 8 h. AU = arbitrary units. (C) Total TAGs from control (Con) or BFA-treated cells starved in nitrogen-deficient medium for 2 days. DMSO and BFA treatment lasted for 3 h. In (b) and (c), three replicates were averaged, and the SEs are shown. Significant differences, as determined by Student’s *t*-test, are indicated by asterisks (*P<0.05, **P<0.01). (D) Fatty acid composition of the TAGs extracted from the solvent control (Con) and BFA-treated cells. Both samples had been starved of nitrogen for one day. Averages from three replicate experiments are shown. No statistically significant differences were found.

We then tested whether BFA treatment of *Chlamydomonas* induces TAG synthesis even in the absence of acetate or nitrogen sources in the medium. *Chlamydomonas CC-503* cells grown in normal TAP (+N, +Ac) medium were collected by centrifugation and resuspended in new TAP medium with or without acetate. After an 8-h treatment with BFA, cells were stained with Nile red and the LD fluorescence was quantified using a TECAN fluorescence spectrophotometer. BFA treatment increased the LD content of cells grown in TAP media either with or without acetate (Fig. 4A). However, BFA induced LD more effectively in the presence of acetate than in its absence: compared to the solvent control, LD fluorescence increased by 40% in the presence of acetate, and by 25% in the absence of acetate.

To determine whether BFA-induced oil accumulation depends on the presence of nitrogen, *CC-503* cells grown in nitrogen-deficient TAP medium for 1 day were treated with BFA for 8 h, and the LD fluorescence was quantified by fluorescence spectrophotometry. Nitrogen depletion induced an increase in fluorescence (Fig. 4B), and BFA treatment further increased LD fluorescence (Fig. 4B). To obtain biochemical evidence of this, total lipids were extracted from *CC-503* cells grown for 2 days under nitrogen starvation conditions and then treated with BFA. Since cells were weak under these growth conditions, BFA treatment lasted only for 3 h in this experiment. TLC and GC analyses revealed that the total TAG content was 19% higher in BFA-treated cells than in control (DMSO-treated) cells under nitrogen starvation (Fig. 4C). However, in contrast to the changes in fatty acid composition induced by BFA under nitrogen-sufficient conditions (Fig. 3C), the fatty acid composition of TAGs was not different between nitrogen-starved cells treated with BFA and the control (Fig. 4D). This result suggests that, in cells treated with BFA under nitrogen-deficient conditions, sources of lipids used to synthesize TAGs are different from those under nitrogen–sufficient conditions.

### BFA Induces ER Stress Marker Gene Expression in *Chlamydomonas*

**Figure 5:**
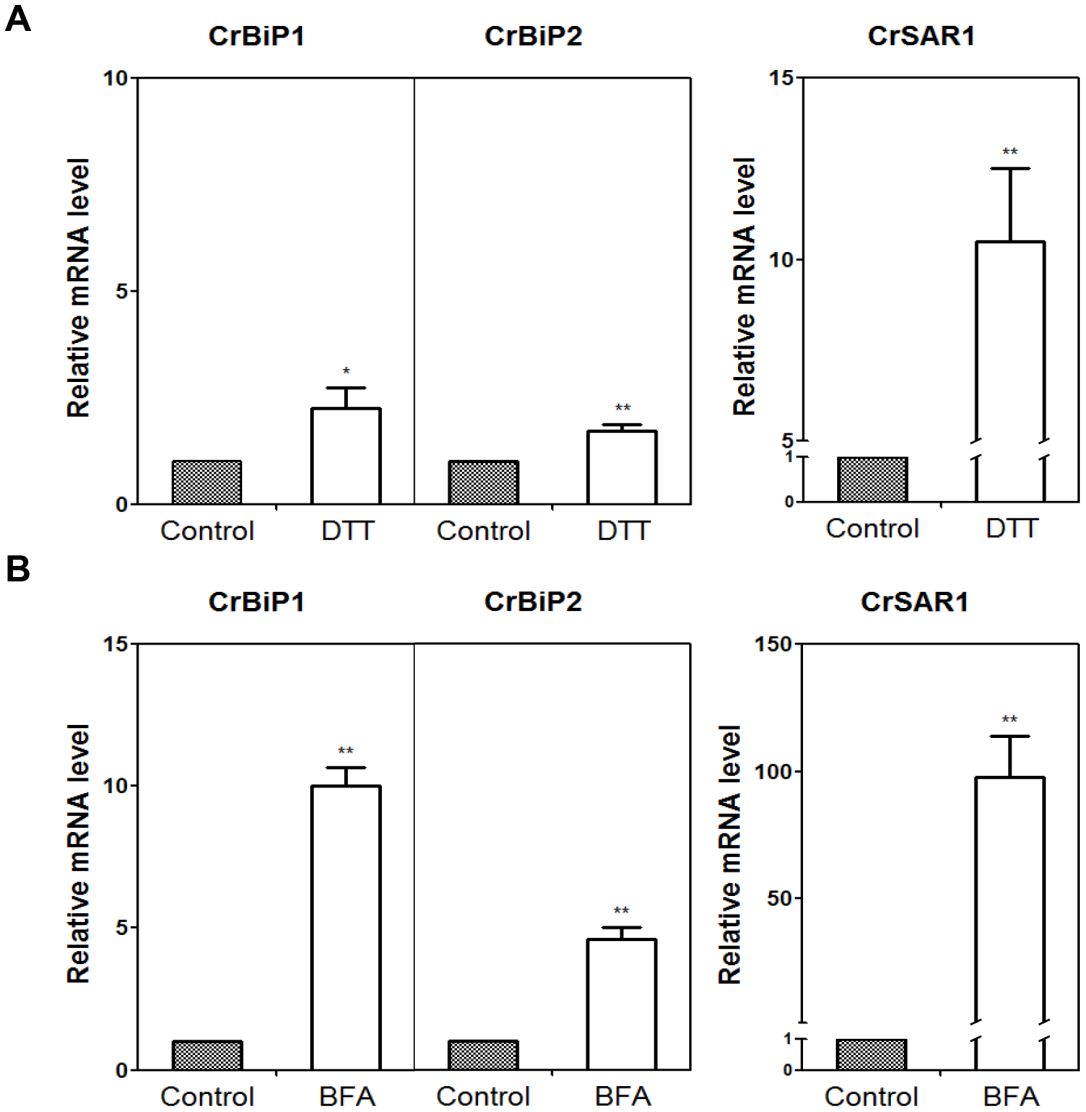
Expression levels of ER stress marker genes in BFA- and DTT-treated *Chlamydomonas reinhardtii CC-503*. Cells in mid-log phase culture were treated with 6 mM DTT dissolved in TAP solution for 2 h, or with BFA (75 µg mL^−1^) dissolved in DMSO for 8 h. Control cells were treated with TAP solution or DMSO, respectively. Transcript levels of BiP homologs (g1475 and Cre02.g080600) and a SAR1 homolog (Cre11.g468300) were analyzed, and fold changes compared with the expression level in the control samples are presented. The averages and standard errors from two independent experiments are shown. Significant differences, as determined by Student’s *t*-test, are indicated by asterisks (*P<0.05, **P<0.01).

To test whether BFA induces ER stress in *Chlamydomonas,* we first searched the *Chlamydomonas* genome for homologs of ER stress marker genes. When the amino acid sequence of AtBiP1, AtBiP2, or AtBiP3 was used in the search, we found that *Chlamydomonas* has two genes with high similarity to AtBiP proteins, and we named these genes *CrBiP1* (g1475) and *CrBiP2* (Cre02.g080600). CrBiP1 and CrBiP2 were 69–71% similar in amino acid sequence to AtBiP1, AtBip2, and AtBiP3 (Fig. S3). A search using the amino acid sequence of AtSAR1 (At1g09180) revealed one similar gene, which we named *CrSAR1* (Cre11.g468300). *CrSAR1* shared 82% amino acid sequence similarity with AtSAR1 (Fig. S4). We then tested whether these genes were induced in *Chlamydomonas* by dithiothreitol (DTT), which has been reported to induce ER stress in many organisms, including *Arabidopsis,* rice, and yeast [26]–[28]. Treatment with 6 mM DTT for 2 h induced the expression of *CrBiP1, CrBiP2,* and *CrSAR1* (Fig. 5A), suggesting that these genes can be used as ER stress marker genes in *Chlamydomonas*, as their homologous genes are used in other organisms [26], [29], [30]. Treatment of the *Chlamydomonas* cells with 75 µg mL^−1^ BFA for 8 h caused a dramatic increase in the expression of *CrBiP1, CrBiP2,* and *CrSAR1* (Fig. 5B).

### BFA Treatment also Induces LD Formation in the Industrially Profitable Alga *Chlorella vulgaris*

**Figure 6:**
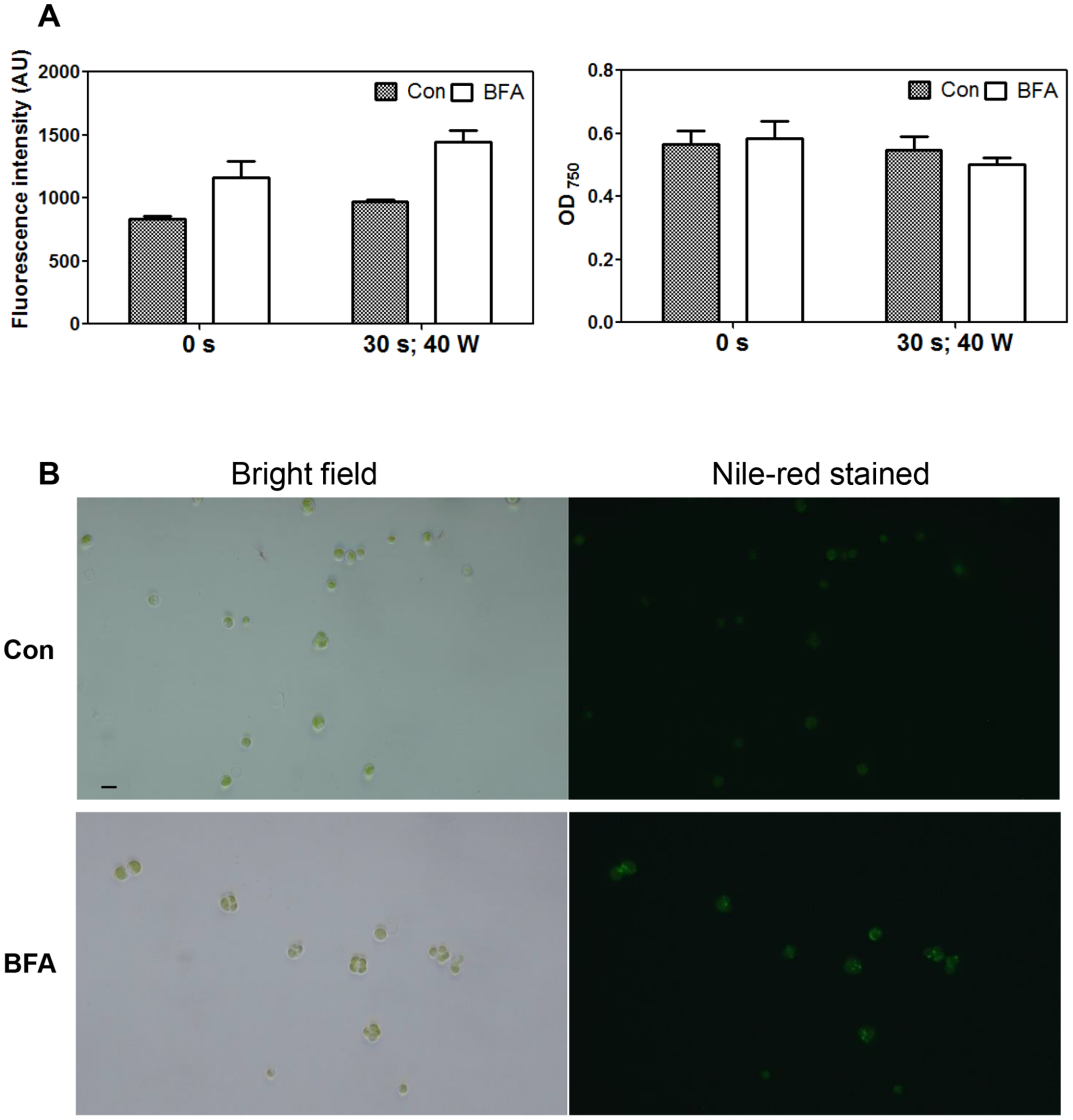
BFA treatment induces LD formation in *Chlorella vulgaris*. (A) A 4 h BFA (75 µg/ml) treatment increases Nile red fluorescence (left) without altering cell density (right). Nile red fluorescence resulting from LDs was quantified using a fluorescence spectrophotometer. AU = arbitrary units. Cells represented by the right-most two bars of each panel were sonicated for 30 s at the 40 watt (W) setting of the sonicator (Vibra Cell™, VC 130PB). Averages from three replicate experiments, and their standard errors are shown. (B) Images of *Chlorella* cells treated with 75 µg mL^−1^ BFA and stained with Nile red. Bar = 10 µm. DMSO was used as a solvent control (Con).

To determine whether our method of LD induction could be applied to industrially profitable algae, we tested the effect of BFA addition on *Chlorella vulgaris*, an industrially cultivated microalga [31]. BFA treatment for 4 h did not significantly alter cell growth (Fig. 6A, right), but effectively induced the formation of LDs (Fig. 6A left, B). Furthermore, the effect was even stronger in cells sonicated for 30 s before BFA treatment (Fig. 6A), presumably because the sonication facilitated drug delivery into the cells.

## Discussion

In the present study, we report that BFA rapidly induces LD formation in two different algae, *Chlamydomonas reinhardtii* (Fig. 1, Fig. S2) and *Chlorella vulgaris* (Fig. 6). The response was greatest during the early log phase of culture (Fig. 2B), and analysis of individual lipid classes revealed that DGTS content decreased, but without altering the total amount of phospholipids and galactolipids (Fig. 3D), suggesting that acyl recycling and assembly into TAGs increased upon BFA treatment. This BFA effect was observed regardless of whether an acetate or nitrogen source was present in the medium or not (Fig. 4).

BFA commonly induces ER stress in many different organisms. We suggest that BFA induces ER stress in *Chlamydomonas* too, because BFA treatment dramatically increased the expression of ER stress marker homologs, *CrBiP1, CrBiP2,* and *CrSAR1* (Fig. 5). Supporting this possibility, DTT, a drug known to induce ER stress in many different organisms [26], [30], [32], also induced the expression of the three genes (Fig. 5). BFA treatment inhibits membrane trafficking from the ER to Golgi membranes, thereby promoting retrograde vesicle transport from Golgi membranes to the ER. This situation might provide ample substrates for the assembly of TAGs, causing LD accumulation in BFA-treated cells. In accordance with this hypothesis, the concentration of DGTS, a lipid synthesized and exported from the ER, was decreased upon BFA treatment (Fig. 3D). Thus, we suggest that DGTS, which is retrieved from other membranes into the ER, is recycled and provides the fatty acid source for the assembly of TAGs in BFA-treated cells. This explanation is further supported by the high level of 18∶3(5,9,12) in the TAGs of BFA-treated cells compared to those of control cells (Fig. 3C). The 18∶3(5,9,12) fatty acid is the major fatty acid present in DGTS, and is rarely found in chloroplastic lipids [25]. However, the increase in TAG levels is much smaller than the decrease in DGTS, and the content of several other lipids was also altered. Thus, BFA seems to cause a serious perturbation in lipid homeostasis, and a part of the degradation product of membrane lipids is converted into TAGs. To accurately determine the sources of fatty acids for BFA-induced TAG synthesis, pulse-chase experiments should be conducted in the future.

To perceive and overcome the ER stress, animal cells invoke pathways that increase the protein folding capacity, decrease the protein production at the ER, and promote lipogenesis [26], [27]. Similar ER stress responses are observed in some plant cells. ER stress activates many lipid metabolic enzymes in maize and soybean cells, and increases TAG accumulation in maize endosperm [29]. Our experiments revealed BFA-induced TAG synthesis in two species of green algae, suggesting that similar alterations in lipid metabolism occur in response to ER stress in green algae. Consistent with this possibility, a homolog of inositol-requiring enzyme 1 (IRE1), a protein kinase/ribonuclease with a central role in the ER stress response, exists in the *Chlamydomonas* genome (g8693 in Phytozome v9.1 at www.phytozome.net). IRE1 is found in yeast, nematodes, fruit flies, plants, and animals, and is thus considered to function in the most ancient pathway of the ER stress response [33]. Further studies of ER stress responses in algae might provide important insight into the evolution of the ER stress response.

**Figure 7:**
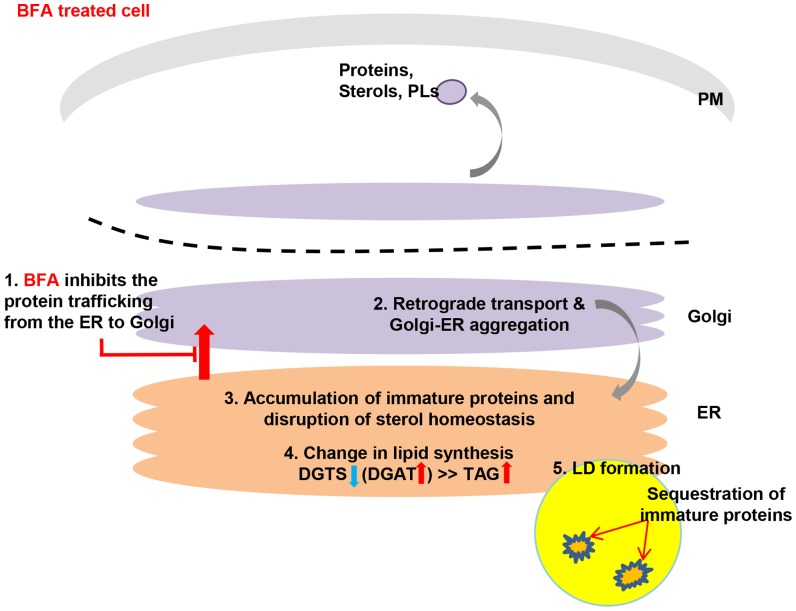
A hypothetical model explaining BFA-induced LD formation. 1. BFA inhibits trafficking of vesicles that deliver proteins and lipids to their final destinations (Lippincott-Schwartz *et al*., Cell, 60 (1990) 821–836). 2. BFA-induced perturbation of vesicle trafficking results in retrograde transport and Golgi-ER aggregation (Lippincott-Schwartz *et al*., Cell, 60 (1990) 821–836). 3. Failure to deliver proteins and lipids to their final destination causes proteins to accumulate (Lippincott-Schwartz *et al*., Cell, 60 (1990) 821–836), and disrupts sterol homeostasis (Stephen M. *et al*., The International Journal of Biochemistry & Cell Biology, 39 (2007) 1843–1851). 4. A portion of the lipids from the disturbed membranes is converted into TAGs, which form LDs. 5. The LDs thus formed may provide a site for sequestering immature proteins, thereby protecting the cell from further damage (Mérigout *et al*., FEBS letters, 518 (2002) 88–92; Fei *et al*., Biochem. J, 424 (2009) 61–67). Since the effect of the drug is rapid, the drug may be a useful trigger for recycling intracellular membranes to TAGs, a lipid form more easily processed to fuel than membrane lipids.

What might be the functions of LDs in ER stress? Fungi [11], [34] and animals [35] were previously found to accumulate LDs in the presence of ER stress. LD formation may be a resistance mechanism against BFA toxicity, which is characterized by the inhibition of overall cell metabolism and protein translation, and the induction of ER stress-associated degradation and lipogenesis [36], which can lead to cell death. It was recently suggested that LDs might serve a protective function for immature proteins upon ER stress [37]. The exposed hydrophobic residues of unfolded proteins tend to aggregate in the cytoplasm and such cytoplasmic aggregates can be extremely toxic to cells. Under such circumstances, the LD core was suggested to provide a safe site for hydrophobic residues of unfolded proteins and might thus reduce or eliminate toxicity [38]. Thus, *Chlamydomonas* may also form lipid droplets to protect itself from the toxicity of denatured proteins resulting from ER stress (Fig. 7).

BFA treatment continuously increased Nile red fluorescence in *Chlamydomonas* for up to 8 h, when the biomass began to decrease (Fig. 1B, C). This decrease in biomass may be due to cell death, since continuous exposure of cells to BFA, which causes ER stress, is highly likely to result in degradation of cells and, consequently, to decreased biomass. It is tempting to speculate that such degradation might involve apoptosis, which has recently been demonstrated in *Chlamydomonas* [39], because severe ER stress leads to apoptosis in many organisms [33]. Interestingly, the effect of BFA on LD accumulation was observed at almost all stages of cell culture, and was greatest during the early log phase (Fig. 2B). This may be because the ER stress responses vary depending on the growth stage of the cell culture, and cells in young culture might respond more vigorously to ER stress than those in older ones.

BFA-induced TAG accumulation was observed before much alteration in chlorophyll content occurred (Fig. S1), indicating that BFA affects lipid accumulation without greatly influencing the chloroplast. Thus, the BFA-induced pathway is, at least in part, different from nitrogen starvation-induced LD formation, which involves degradation of chloroplastic membranes [7].

Even under acetate-depleted conditions, BFA induced LD formation (Fig. 4A). This is not surprising if the sources of the acyl-CoA pool used for TAG synthesis are pre-existing ER or other membrane lipids. Indeed, the concentration of DGTS, a lipid derived and trafficked from the ER of *Chlamydomonas*, decreased upon BFA treatment (Fig. 3D), supporting our explanation. However, we cannot exclude the possibility that newly synthesized acyl-CoAs, which use acetate in the medium as substrate, are also used for BFA-induced LD formation, since the effect of BFA was enhanced when acetate was added to the medium (Fig. 4A). Thus, the acyl-CoA pool used for BFA-induced TAG synthesis seems to be made not only from recycled DGTS, but also from fatty acids synthesized *de novo* from acetate.

Surprisingly, even under nitrogen depletion, when cells already accumulate LDs, BFA further increases TAG content (Fig. 4B, C). In cells starved of nitrogen and then treated with BFA, a large proportion of TAGs would already have been synthesized in chloroplasts, but a small portion of TAGs, in addition, might have been synthesized at the ER upon BFA treatment. Under nitrogen-deficient conditions, *de novo* fatty acid synthesis increases [6], [7]. Thus, newly synthesized fatty acids, rather than recycled fatty acids, may be the major substrate of BFA-induced TAG formation under nitrogen-deficient conditions. In accordance with this explanation, the fatty acid composition of TAGs was not different between BFA-treated and control cells under nitrogen starvation conditions (Fig. 4D). This was in contrast to the fatty acid composition of TAGs under nitrogen-sufficient conditions (Fig. 3C), indicating that different sources of acyl-CoAs are used for most of the TAG synthesis under nitrogen-deficient and nitrogen-sufficient conditions.

Previous approaches to increase TAG content in *Chlamydomonas* focused on increasing the activity of enzymes involved in TAG biosynthesis [40], or disrupting starch synthesis [5]. However, both strategies depend on nitrogen starvation or other stress conditions that require long-term treatment and are expensive. Here, we demonstrated that BFA rapidly induces LD formation in two microalgal species, *Chlamydomonas* and *Chlorella.* It is particularly interesting that the drug was effective regardless of whether or not an exogenous acetate or nitrogen source was present in the growth medium (Fig. 4). Therefore, the drug can be used to convert membrane lipids into TAGs after harvest of biomass grown under optimal conditions. This is valuable, since neutral lipids such as TAGs are much easier to convert into fuel than membrane lipids. However, the problem in this scheme is that the drug is non-specific and has pleiotropic effects, including protein degradation and, in the case of prolonged treatment, cell death. Thus, it is necessary to pinpoint specifically which aspect of the BFA response alters the lipid content and to use this knowledge to reduce the amount of time needed to produce LDs and increase the portion of neutral lipids harvestable from the algae. Further studies of the detailed mechanisms by which BFA affects lipid metabolism may provide a clue for developing an efficient strategy for microalgae–based biofuel production.

## Supporting Information

Figure S1 **BFA does not significantly change chlorophyll content.** *Chlamydomonas reinhardtii* strain *CC-503* cells at the mid-log phase (48 h after the beginning of sub-culture) were treated with 75 µg mL^−1^ BFA for 8 h, and then chlorophyll content was measured.
(TIF)

Figure S2 **BFA induces LD formation in the** ***CC-125*** **line of** ***Chlamydomonas reinhardtii*** **, which has a cell wall.** To facilitate BFA uptake, samples were sonicated for 0, 10, or 30 s before BFA treatment. All images were captured in the FITC channel, except the bottom right image, which was captured in the TRITC channel.
(TIF)

Figure S3 **Multiple sequence alignment of BiP orthologs from** ***Arabidopsis*** **,** ***Chlamydomonas*** **, and** ***Saccharomyces*** **.** CLUSTALW (http://www.genome.jp/tools/clustalw) was used for the alignment. Stars indicate conserved amino acids.
(DOCX)

Figure S4 **Multiple sequence alignment of SAR1 orthologs from** ***Arabidopsis*** **,** ***Chlamydomonas*** **, and** ***Saccharomyces*** **.** CLUSTALW (http://www.genome.jp/tools/clustalw) was used for the alignment. Stars indicate conserved amino acids.
(TIFF)
