# Supplementary figures and images for: Rapid Induction of Lipid Droplets in Chlamydomonas reinhardtii and Chlorella vulgaris by Brefeldin A
Source: PLoS One. 2013 Dec 13;8(12):e81978. doi: 10.1371/journal.pone.0081978 (PMC3862487; doi:10.1371/journal.pone.0081978)

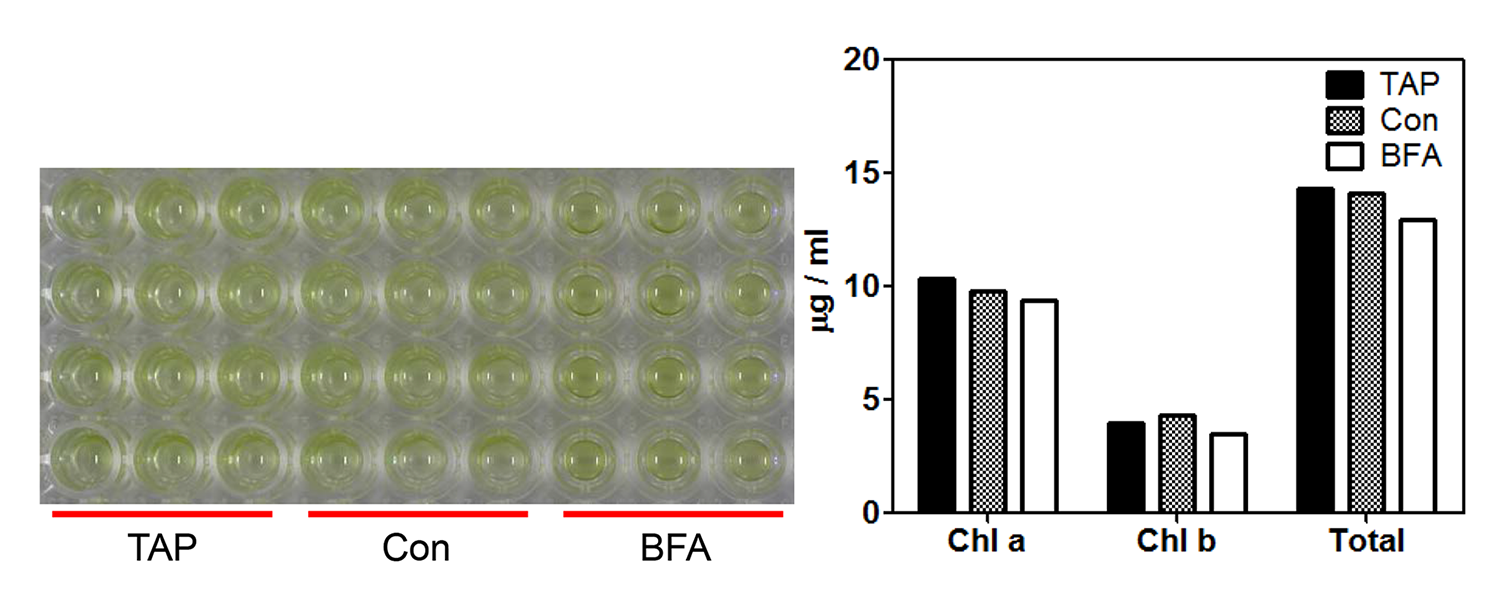

Supplement: Figure S1 — BFA does not significantly change chlorophyll content. Chlamydomonas reinhardtii strain CC-503 cells at the mid-log phase (48 h after the beginning of sub-culture) were treated with 75 µg mL−1 BFA for 8 h, and then chlorophyll content was measured. (TIF) [file pone.0081978.s001.tif]

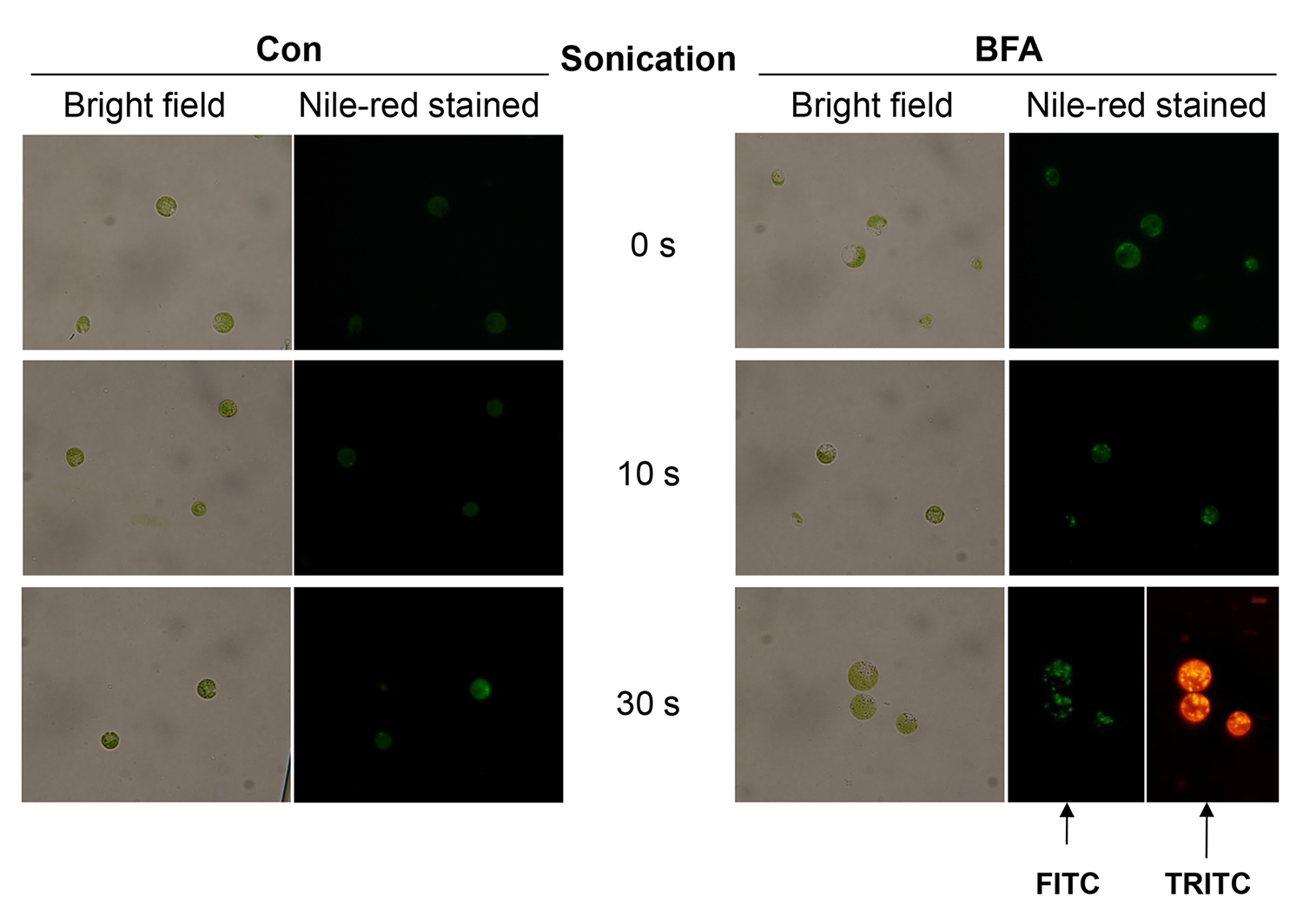

Supplement: Figure S2 — BFA induces LD formation in the CC-125 line of Chlamydomonas reinhardtii , which has a cell wall. To facilitate BFA uptake, samples were sonicated for 0, 10, or 30 s before BFA treatment. All images were captured in the FITC channel, except the bottom right image, which was captured in the TRITC channel. (TIF) [file pone.0081978.s002.tif]

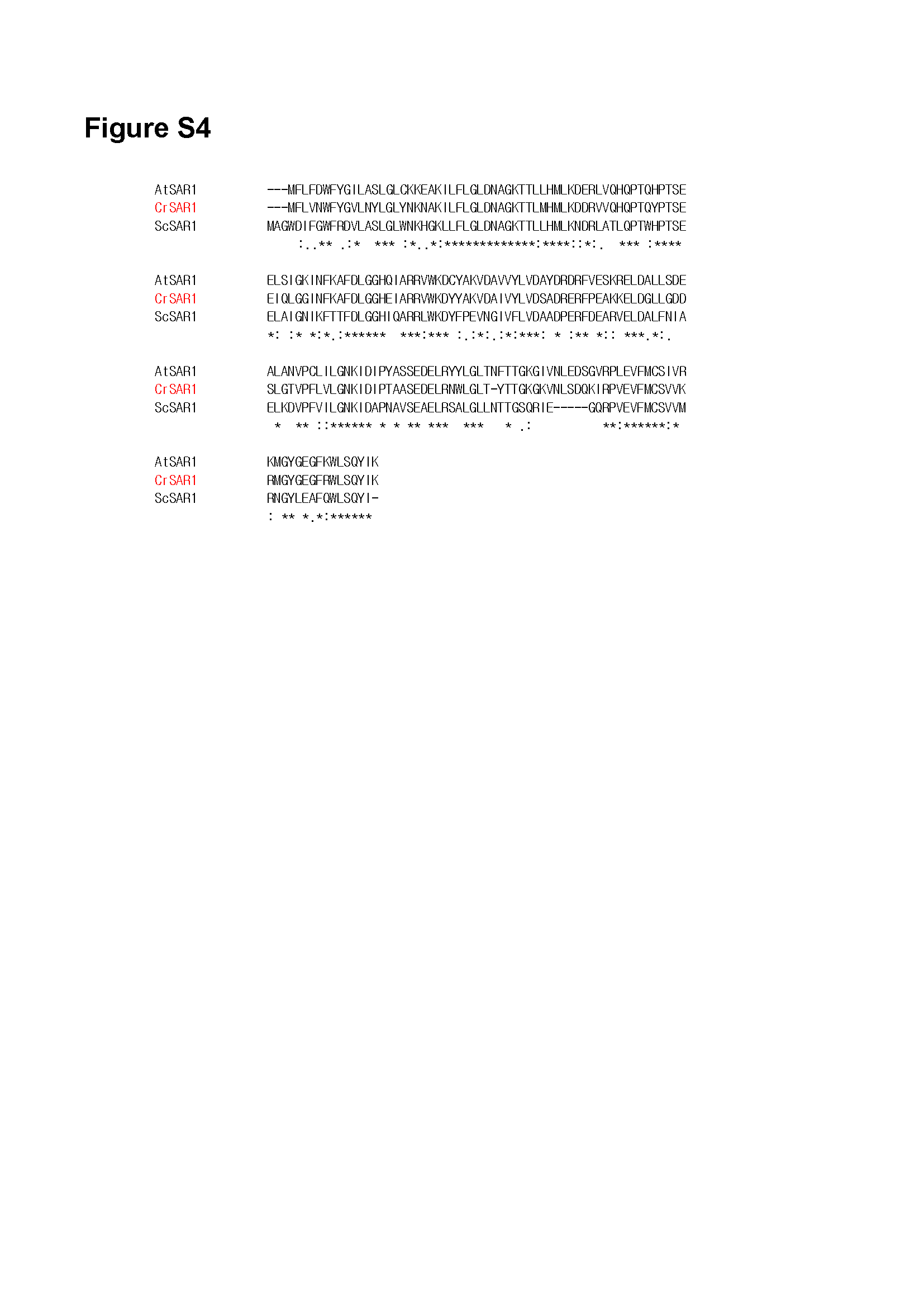

Supplement: Figure S4 — Multiple sequence alignment of SAR1 orthologs from Arabidopsis , Chlamydomonas , and Saccharomyces . CLUSTALW (http://www.genome.jp/tools/clustalw) was used for the alignment. Stars indicate conserved amino acids. (TIFF) [file pone.0081978.s004.tiff]
