# Supplementary material for: Rapid Induction of Lipid Droplets in Chlamydomonas reinhardtii and Chlorella vulgaris by Brefeldin A
Source: PLoS One. 2013 Dec 13;8(12):e81978. doi: 10.1371/journal.pone.0081978 (PMC3862487; doi:10.1371/journal.pone.0081978)
Supplement: Figure S3 — Multiple sequence alignment of BiP orthologs from Arabidopsis , Chlamydomonas , and Saccharomyces . CLUSTALW (http://www.genome.jp/tools/clustalw) was used for the alignment. Stars indicate conserved amino acids. (DOCX) [file pone.0081978.s003.docx]

**Figure S3**

AtBiP1 ----------------------MARSFGANSTVVLAIIFFGCLFALSSAIEEATKLGSVI

AtBiP2 ----------------------MARSFGANSTVVLAIIFFGCLFAFSTAKEEATKLGSVI

AtBiP3 -------MIFIKENTAKMTRNKAIACLVFLTVLDFLMNIGAALMSSLAIEGEEQKLGTVI

CrBiP1 ---------------------------MAQWKAAVLLLALACASYGFGVWAEEEKLGTVI

CrBiP2 MANSLKTRKFHTSTGHHGVTRRLCMVGLGLIATLVAASALASIPQAKAASPTTDKLGTVI

ScKAR2 -------MFFNRLSAGKLLVPLSVVLYALFVVILPLQNSFHSSNVLVRGADDVENYGTVI

. : *:**

AtBiP1 GIDLGTTYSCVGVYKNGHVEIIANDQGNRITPSWVGFTDSERLIGEAAKNQAAVNPERTV

AtBiP2 GIDLGTTYSCVGVYKNGHVEIIANDQGNRITPSWVGFTDSERLIGEAAKNQAAVNPERTV

AtBiP3 GIDLGTTYSCVGVYHNKHVEIIANDQGNRITPSWVAFTDTERLIGEAAKNQAAKNPERTI

CrBiP1 GIDLGTTYSCVGVYKNGRVEIIANDQGNRITPSYVAFTDEERLIGDAAKNQATVNPKRTV

CrBiP2 GIDLGTTYSCVGVYKNGRVEIIANDQGNRITPSYVAFTDEERLIGDAAKNQATVNPKRTI

ScKAR2 GIDLGTTYSCVAVMKNGKTEILANEQGNRITPSYVAFTDDERLIGDAAKNQVAANPQNTI

***********.* :* :.**:**:********:*.*** *****:*****.: **:.*:

AtBiP1 FDVKRLIGRKFEDKEVQKDRKLVPYQIVNKDGKPYIQVKIKDGETKVFSPEEISAMILTK

AtBiP2 FDVKRLIGRKFEDKEVQKDRKLVPYQIVNKDGKPYIQVKIKDGETKVFSPEEISAMILTK

AtBiP3 FDPKRLIGRKFDDPDVQRDIKFLPYKVVNKDGKPYIQVKVK-GEEKLFSPEEISAMILTK

CrBiP1 YDVKRLIGRKYEDKEVQRDKKLVSYDIVDRQGKPYVAVDVK-GEQKVFSPEEISAMILQK

CrBiP2 YDVKRLIGRKFSDADVQRDRKLVSYDIVDRQGKPYVAVDVK-GEQKVFSPEEISAMILQK

ScKAR2 FDIKRLIGLKYNDRSVQKDIKHLPFNVVNKDGKPAVEVSVK-GEKKVFTPEEISGMILGK

:* ***** *:.* .**:* * :.:.:*:::*** : *.:* ** *:*:*****.*** *

AtBiP1 MKETAEAYLGKKIKDAVVTVPAYFNDAQRQATKDAGVIAGLNVARIINEPTAAAIAYGLD

AtBiP2 MKETAEAYLGKKIKDAVVTVPAYFNDAQRQATKDAGVIAGLNVARIINEPTAAAIAYGLD

AtBiP3 MKETAEAFLGKKIKDAVITVPAYFNDAQRQATKDAGAIAGLNVVRIINEPTGAAIAYGLD

CrBiP1 MKDTAEAYLGKTVKHAVVTVPAYFNDAQRQATKDAGTISGLNVVRIINEPTAAAIAYGLD

CrBiP2 MKDTAEAYLGKTVKHAVVTVPAYFNDAQRQATKDAGTISGLNVVRIINEPTAAAIAYGLD

ScKAR2 MKQIAEDYLGTKVTHAVVTVPAYFNDAQRQATKDAGTIAGLNVLRIVNEPTAAAIAYGLD

**: ** :**..:..**:******************.*:**** **:****.********

AtBiP1 KKGGEKNILVFDLGGGTFDVSVLTIDNGVFEVLSTNGDTHLGGEDFDHRVMEYFIKLIKK

AtBiP2 KKGGEKNILVFDLGGGTFDVSVLTIDNGVFEVLSTNGDTHLGGEDFDHRIMEYFIKLIKK

AtBiP3 KKGGESNILVYDLGGGTFDVSILTIDNGVFEVLSTSGDTHLGGEDFDHRVMDYFIKLVKK

CrBiP1 KKGGEKNILVFDLGGGTFDVSILTIDNGVFEVISTNGDTHLGGEDFDQRVMEYFIKLIKK

CrBiP2 KKGGEKNILVFDLGGGTFDVSILTIDNGVFEVISTNGDTHLGGEDFDQRVMEYFIKLIKK

ScKAR2 KSDKEHQIIVYDLGGGTFDVSLLSIENGVFEVQATSGDTHLGGEDFDYKIVRQLIKAFKK

*.. * :*:*:**********:*:*:****** :*.*********** ::: :** .**

AtBiP1 KHQKDISKDNKALGKLRRECERAKRALSSQHQVRVEIESLFDGVDFSEPLTRARFEELNN

AtBiP2 KHQKDISKDNKALGKLRRECERAKRALSSQHQVRVEIESLFDGVDLSEPLTRARFEELNN

AtBiP3 KYNKDISKDHKALGKLRRECELAKRSLSNQHQVRVEIESLFDGVDFSEPLTRARFEELNM

CrBiP1 KYKKDISGDARALQKLRREAERAKRALSSQHQVRVEIEALYEGIDLSEPLTRARFEELNM

CrBiP2 KYKKDISGDARALQKLRREAERAKRALSSQHQVRVEIEALYEGIDLSEPLTRARFEELNM

ScKAR2 KHGIDVSDNNKALAKLKREAEKAKRALSSQMSTRIEIDSFVDGIDLSETLTRAKFEELNL

*: *:* : :** **:**.* ***:**.* ..*:**::: :*:*:**.****:*****

AtBiP1 DLFRKTMGPVKKAMDDAGLQKSQIDEIVLVGGSTRIPKVQQLLKDFFEGKEPNKGVNPDE

AtBiP2 DLFRKTMGPVKKAMDDAGLQKSQIDEIVLVGGSTRIPKVQQLLKDFFEGKEPNKGVNPDE

AtBiP3 DLFKKTMEPVKKALKDAGLKKSDIDEIVLVGGSTRIPKVQQMLKDFFDGKEPSKGTNPDE

CrBiP1 DLFKKTMGPVKKAMDDANLKKTEIDEIVLVGGSTRIPKVQDLLREWFDGKEPNKGVNPDE

CrBiP2 DLFKKTMGPVKKAMDDANLKKTEIDEIVLVGGSTRIPKVQDLLREWFGGKEPNKGVNPDE

ScKAR2 DLFKKTLKPVEKVLQDSGLEKKDVDDIVLVGGSTRIPKVQQLLESYFDGKKASKGINPDE

***:**: **:*.:.*:.*:*.::*:**************::*..:* **:..** ****

AtBiP1 AVAYGAAVQGGILSGEGGDETKDILLLDVAPLTLGIETVGGVMTKLIPRNTVIPTKKSQV

AtBiP2 AVAYGAAVQGGILSGEGGDETKDILLLDVAPLTLGIETVGGVMTKLIPRNTVIPTKKSQV

AtBiP3 AVAYGAAVQGGVLSGEGGEETQNILLLDVAPLSLGIETVGGVMTNIIPRNTVIPTKKSQV

CrBiP1 AVAYGAAVQGGILGGEGGDEVKDILLLDVAPLSLGIETVGGVMTKLIPRNTVIPTKKSQT

CrBiP2 AVAYGAAVQGAILSGEEEESTEGLIVIDRTPLSLGIETTGGVMTNLIPRNSVIPTKKSQT

ScKAR2 AVAYGAAVQAGVLSGEEG--VEDIVLLDVNALTLGIETTGGVMTPLIKRNTAIPTKKSQI

*********..:*.** .:.::::* .*:*****.***** :* **:.*******

AtBiP1 FTTYQDQQTTVSIQVFEGERSLTKDCRLLGKFDLNGIPPAPRGTPQIEVTFEVDANGILN

AtBiP2 FTTYQDQQTTVSIQVFEGERSLTKDCRLLGKFDLTGVPPAPRGTPQIEVTFEVDANGILN

AtBiP3 FTTYQDQQTTVTINVYEGERSMTKDNRELGKFDLTGILPAPRGVPQIEVTFEVDANGILQ

CrBiP1 FTTYQDQQTTVSIQVYEGERAMTKDNHKLGQFDLNGIPPAPRGTPQIEVTFEVDANGILN

CrBiP2 FSTAADNQPTVSIQVYEGERALTKDNHKLGQFDLNGIPPAPRGTPQIEVTFEVDANGILT

ScKAR2 FSTAVDNQPTVMIKVYEGERAMSKDNNLLGKFELTGIPPAPRGVPQIEVTFALDANGILK

*:* *:*.** *:*:****:::** . **:*:*.*: *****.******* :******

AtBiP1 VKAEDKASGKSEKITITNEKGRLSQEEIDRMVKEAEEFAEEDKKVKEKIDARNALETYVY

AtBiP2 VKAEDKASGKSEKITITNEKGRLSQEEIDRMVKEAEEFAEEDKKVKEKIDARNALETYVY

AtBiP3 VKAEDKVAKTSQSITITNDKGRLTEEEIEEMIREAEEFAEEDKIMKEKIDARNKLETYVY

CrBiP1 VAAEDKGTGKKEKITITAEKGRLSQDDIERMVKEAEEFAEQDKAVKAKIDARNQLETYCY

CrBiP2 VSAQDKGTGKKEKITITAEKGRLSQDDIERMVKEAEEFAEQDKAVKAKIDARNQLETYCY

ScKAR2 VSATDKGTGKSESITITNDKGRLTQEEIDRMVEEAEKFASEDASIKAKVESRNKLENYAH

* * ** : ..:.**** :****::::*:.*:.***:**.:* :* *:::** **.* :

AtBiP1 NMKNQVNDKDKLADKLEGDEKEKIEAATKEALEWLDE-NQNSEKEEYDEKLKEVEAVCNP

AtBiP2 NMKNQVSDKDKLADKLEGDEKEKIEAATKEALEWLDE-NQNSEKEEYDEKLKEVEAVCNP

AtBiP3 NMKSTVADKEKLAKKISDEDKEKMEGVLKEALEWLEE-NVNAEKEDYDEKLKEVELVCDP

CrBiP1 NMKSTVED--KMKDKIEEEDKEKITAAVKEALEWLDE-NPDAEPDEYKDKLKEVEDVCNP

CrBiP2 NMKNTVED--KMKDKIEEEDKEKITAAVKEALEWLDE-NPDADTSEYKDRLKEVEDVCNP

ScKAR2 SLKNQVNG--DLGEKLEEEDKETLLDAANDVLEWLDDNFETAIAEDFDEKFESLSKVAYP

.:*. * . .: .*:. ::**.: . ::.****:: : .::.::::.:. *. *

AtBiP1 IITAVYQRSGGAPGGAGGESSTEEEDESHDEL--

AtBiP2 IITAVYQRSGGAPG-AGGESSTEEEDESHDEL--

AtBiP3 VIKSVYEKTEGENEDDDGDDHDEL----------

CrBiP1 IIAEVYKKSGGPSDGGDSE---DLGDHDEL----

CrBiP2 IIAEVYKKSGGPSGGGDSHEDEDLADHDEL----

ScKAR2 ITSKLYGGADGSGAADYDDEDEDDDGDYFEHDEL

: :* : * .. :
